# Multimodal Detection of Pain and Anticipation Anxiety from Ultra-Short Duration Wearable Sensors Measurements

**DOI:** 10.3390/s26103181

**Published:** 2026-05-18

**Authors:** Andrew G. Peitzsch, Katie Geary, Youngsun Kong, Hugo Posada-Quintero, Drew Havard, William R. D’Angelo, Ki H. Chon

**Affiliations:** 1 Biomedical Engineering Department, University of Connecticut, Storrs, CT 06269, USA; 2 Naval Medical Research Unit San Antonio, United States Navy, JBSA Fort Sam Houston, San Antonio, TX 78234, USA

**Keywords:** wearable devices, electrodermal activity, photoplethysmography, machine learning, pain and anxiety, dental health

## Abstract

With the continued rise in outpatient surgical procedures, modern medicine requires more advanced tools for pain and anxiety monitoring and management. The current standard of care requires patient responses on visual analog scales, which may be subjective and are difficult to assess when a subject is unresponsive. Electrodermal activity (EDA) and pulse rate variability (PRV), two non-invasive, wearable, and objective measurements of sympathetic nervous system activity, can help provide insight into a patient’s psychological or emotional state without user input, allowing for continued monitoring even when a patient is unable to respond. However, methods based on these measurements have largely been relegated to longer duration (>60 s) or post hoc analysis, which does not suit the needs of medical care environments. Here we propose new methods for handling ultra-short (<10 s) signals to allow rapid evaluation of pain and anxiety state. We show how machine learning models trained on these signals can obtain high degrees of classification performance (AUC > 0.88) between no pain or anxiety and medium or higher pain and anxiety on signals obtained during two different forms of painful stimulation. We also show how these signals can measure the degree of stimulation irrespective of perceived pain from the patient. Further development of these algorithms will allow for greater monitoring and control of patient comfort in a clinical setting.

## 1. Introduction

Advancements in anesthetics have allowed for rapid growth in the field of conscious outpatient surgery which yields many benefits in cost, convenience, and surgical outcomes [1]. Dental procedures, a long-standing form of conscious surgery, are a prime example of how with wakefulness comes the ability for patients to experience spikes and lulls in anxiety and pain throughout a procedure [2,3]. Literature has shown that anxiety before and during these wakeful procedures can have negative impact on the patient’s intraoperative pain, recovery times, and overall outcomes [4,5,6,7]. Importantly, untreated pre- and intraoperative anxiety has been linked to higher propofol and opioid requirements during the induction and maintenance of anesthesia, adding unnecessary costs to procedures and potentially further increasing anxiety, creating a vicious cycle [8,9]. It is vitally important that surgeons track patient anxiety and pain both before and during procedures to ensure comfort and success of the operation.

Current methods for determining patient pain and anxiety primarily rely on verbal interaction with patients or observing physical reactions. Visual analog scales (VAS), like the Defense and Veterans Pain Rating Scale, have been developed to facilitate consistent rating of pain and anxiety, but are still open to subjectivity and of limited use when the patient cannot communicate their rating [10,11]. This issue commonly arises in clinical and research settings, including during dental procedures and in procedures where the patient is sedated or incapacitated [10]. Observation-based scoring is the current standard for pain monitoring in sedated or incapacitated patients but is not as accurate as VAS-based methods and has led to systemic issues such as care providers underestimating pain and associated anesthetic requirements [12,13]. Observation-based scoring is limited for surgical anxiety, often measuring agitation or alertness instead [14,15]. Neither VAS nor observational scores can provide continuous-time tracking and provide alerts for increases in pain or anxiety levels. A system capable of tracking and communicating patients’ pain and anxiety levels during procedures from physiological signals would greatly benefit both practitioners and patients, providing a consistent and objective measurement of patient status to inform treatment.

Pain stimuli travel along the C and Aδ nerve fiber afferents to dorsal root and trigeminal ganglia, from which the signal travels to the “pain matrix” of the brain where it is processed [16]. Here, the stimulus elicits a response that raises sympathetic nervous system (SNS) activity and lowers parasympathetic nervous system (PNS) activity [17]. Pain is processed for emotional responses in the amygdala, with can further modulate pain expression throughout the body. Pain anticipation anxiety is known to play a role in exacerbating the sensation of pain through action in the amygdala and further effects the response of the SNS [17,18]. Anxiety due to pain anticipation has been determined to follow similar pathways to the experienced pain, and as such both may be detected using quantitative measurements of SNS activity [18].

Non-invasive measurement of SNS and PNS activity is commonly done through changes in heart rhythms. Heart rate is primarily controlled by the ANS, with the sympathetic and parasympathetic nervous systems competing to raise and lower the rate, respectively. Pulse rate variability (PRV), a measurement of the change in heart rate using photoplethysmographic (PPG) pulses, can reveal information about the interactions of the underlying two systems. PRV showed strong correlation with heart rate variability and is often measured through statistical features such as the range, mean, and standard deviation [19,20]. Frequency features of PRV can be computed for longer measurements, giving a better measurement of sympathetic and parasympathetic activity individually [21].

SNS activity can also be measured using electrodermal activity (EDA), or skin conductance. Eccrine sweat glands are highly innervated by post-ganglionic sudomotor nerves of the SNS, allowing the glands to open or close in response to autonomic nervous system activation [22]. This behavior is primarily directed towards homeostasis and thermoregulation over the long term, but short-term activation of eccrine sweat glands is primarily controlled through the sympathetic nervous pathway, allowing short-term and rapid control in response to individual stimuli such as stress or emotions [23]. EDA supplies a microcurrent across the skin to measure changes in conductance caused by this change in sweat response, allowing a faster measurement of SNS activity compared to PRV [23,24,25]. The hands and feet are known to have high eccrine sweat gland density, allowing for easy measurement from wearable devices [24,26].

Recent work has found that pain and stress response is primarily limited to EDA frequencies between 0.08 and 0.24 Hz [27]. Using Variable Frequency Complex Demodulation [28], isolation of these frequencies in a tight signal band was completed before reconstructing the frequencies into a continuous signal called TVSymp [29]. Modified TVSymp (MTVSymp), defined as the TVSymp value above the mean of the last 5 s, to extract time-limited increases in TVSymp, was developed to expand upon this [25]. These decomposition methods are able to detect large changes in EDA associated with stress while reducing the noise from movement artifacts, allowing EDA signals to be processed in a more stable, reproducible and sensitive manner [21,30]. EDA has previously been employed to monitor anxiety, including cases in a dental setting [31,32,33]. Additionally, EDA data has been shown to contain sufficient biomarkers to employ machine learning to autonomously detect patient anxiety [33,34,35,36,37]. However, these studies have largely been focused on social or general anxiety disorders rather than on pain anticipation anxiety. Prior research has found that EDA is affected by pain anticipation, potentially biasing pain detection methods, and that situational and context clues such as the circumstances a subject is in that may be causing emotional arousal can increase anxiety detection performance [38,39].

EKG-derived heart rate variability (HRV) and PRV signals are typically analyzed over long durations (several minutes to hours) to detect anxiety [33,36,40]. Some research has been done on so-called ultra-short HRV (<60 s), but this has not been investigated in the more common PPG sensors used in wearable devices [33,40,41]. Short-duration EDA signals (<30 s) have been used to detect the presence of stress, but not anxiety [42]. The traditional approaches using long-duration PRV and EDA signals do not provide the resolution required to properly detect the onset of pain or anxiety in a clinical setting.

In this paper, we present an approach to detecting both pain and pain anticipation anxiety in near-real time from ultra-short duration measurements of EDA and PPG from wearable devices. Thunberg’s Thermal Grill illusion (TGI) and transcutaneous electrical nervous stimulation (TENS) were used to produce both pain and pain anticipation anxiety, while a wearable device was used to record physiological measures. We compare features extracted from these signals and their classification importance to the final trained machine learning models. We also compare several machine learning model types to assess optimal model performance.

## 2. Methods

### 2.1. Subjects

A total of 82 healthy subjects (54 males, 28 females) from the Joint Base San Antonio (JBSA) area between the ages of 18 and 90 were recruited without regard to ethnicity. Eighty were admitted to the study and completed all rounds of testing. Subjects were excluded from the study if any of the following conditions were met:

- chronic or acute nerve pain,
- peripheral neuropathy,
- Raynaud’s disease,
- epilepsy,
- a history of heart issues or problems (suspected or diagnosed),
- cardiovascular disease,
- an implanted electronic device,
- the use of any drugs that might affect the perception of pain (i.e., opioids (narcotics), benzodiazepines (valium), anticonvulsants (gabapentin, Neurontin, etc.), or Tricyclic Antidepressants (TCAs)),
- swollen, infected, inflamed skin and open burns or wounds on the hands or forearms where equipment would be placed.

### 2.2. Pain Modalities

During testing, the participants completed two tests; the first being a series of Thermal Grill Illusion tests, followed by exposure to a series of intensity levels from a Transcutaneous Electronic Nervous Stimulation machine.

#### 2.2.1. Thunberg’s Thermal Grill Illusion (TGI)

**Figure 1:**
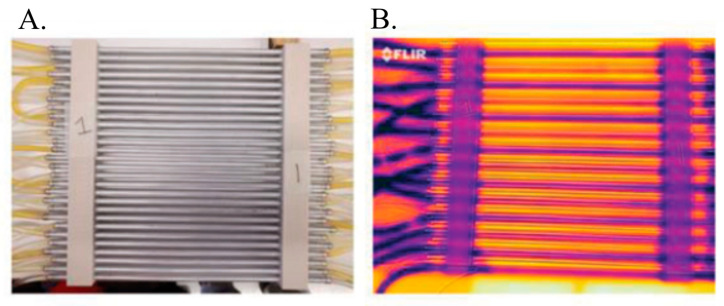
Thermal Grill used for TGI stimulation imaged in (**A**) visible and (**B**) infrared to show temperature. The cold water temperature was set to 18 °C, except for the sham where it was replaced with the warm water, and the warm water temperature was varied between 37 °C and 40 °C based on the stimulation level.

Thunberg’s Thermal Grill illusion (TGI) is a sensory illusion caused by simultaneous exposure of the skin to warm and cold [43]. The intensity of this illusion can be altered by increasing or decreasing the temperature gradient between the hot and cold stimuli. For this study, three levels were created; (1) Level 0 (sham)—All tubes circulating water at 35 °C, (2) Level 1—alternating rods containing ice water (0–10 °C) and warm water (37 °C) and (3) Level 2—alternating rods containing ice water (0–10 °C) and hot water (40 °C). The setup is shown in Figure 1.

#### 2.2.2. Transcutaneous Electrical Nervous Stimulation (TENS)

Transcutaneous electrical nervous stimulation (TENS) uses small electrodes to deliver electrical impulses that flood the nervous system and can scramble pain signals before reaching the spinal cord or brain in patients with chronic pain [44]. However, these systems also tend to cause muscle spasms that can be uncomfortable to healthy patients. The intensity of this sensation can be changed by increasing or decreasing the frequency and/or current delivered to the skin. For this study, five levels were created by varying the frequency from 0 Hz (off, sham) to 100 Hz in uniform increments on the ElfCare TENS device (Mediseb Ltd., Herzliya, Israel). To improve skin conductance and prevent overheating of the skin, an ultrasound gel was used for each subject and reapplied during rounds as needed.

### 2.3. Sensors

Participants were fitted with the Shimmer3 GSR Consensys system (Shimmer Research Ltd., Dublin, Ireland) which includes two finger electrodes that generate the EDA, and a wrist strap and optical pulse sensor that generates a photoplethysmogram (PPG). EDA and PPG data from the Shimmer3 GSR+ system were recorded and monitored in real time. All subjects were asked to wash their hands with soap and warm water for 30 s before placing the shimmer on their left hand for each of the two tests.

### 2.4. Experiment Protocol

The following human subject research was reviewed and approved by the IRB for the Naval Medical Research Unit San Antonio (NAMRUSA) under protocol NAMRUSA2020.006.

**Figure 2:**
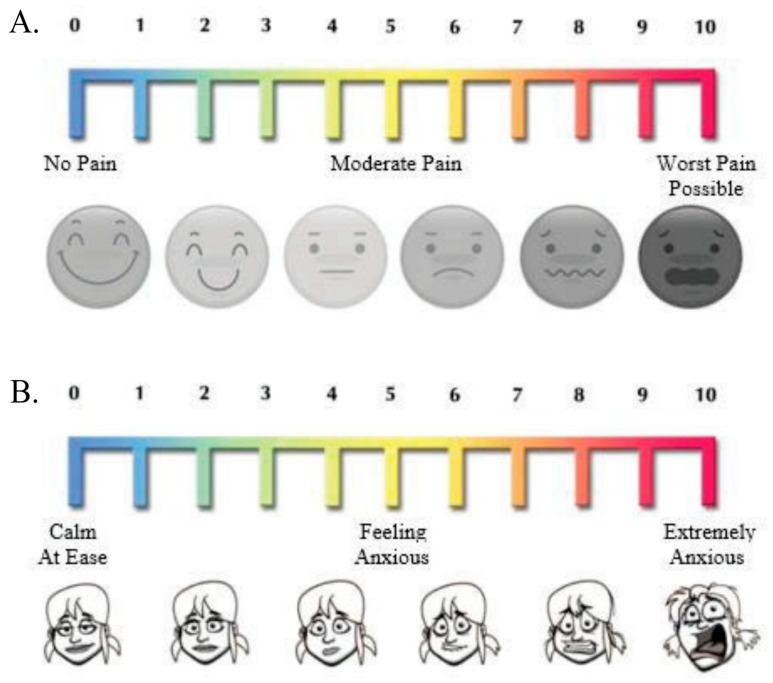
VAS for (**A**) pain and (**B**) anxiety. The subjects selected a numeric score with facial expressions providing references for comparison.

Before and after each round of stimulation, subjects were given a response form with two Visual Analog Scales (VAS); one for anxiety (completed pre-exposure) and one for pain (completed post-exposure) shown in Figure 2. The Shimmer system was set to record continuously throughout all rounds of testing. For each subject, the system was collecting electrodermal activity (EDA) and photoplethysomography (PPG) data via two EDA sensors located on the middle and forefinger and a PPG sensor at the base of the thumb. Subjects were informed during the instruction section that they could quit or stop the test at any time, but that at least five seconds of data was required for the data to be used.

The order of the three levels was held consistent for each subject but made to appear random. Subjects were exposed to Level 0, 2, and 1 in that order. A 10 s countdown clock was displayed to prepare subjects for exposure and induce anxiety through anticipation of pain. The color of the slides progressed from red, to yellow, to green as the countdown approached zero. Following a buzzer signifying the start of the test and when the subject should place their hand on the grill, a second 10 s countdown in white appeared to signify the length of the trial and ended with another buzzer. Following the second buzzer, a screen appeared telling subjects to remove their right hand from the stimulus but to keep their left hand, with the Shimmer, still for an additional 10 s to collect ramp-down data. This process was completed for each of the three levels, with a 30 s recovery period between each round.

As with the TGI testing, the order of the TENS levels delivered was held consistent from subject to subject, but made to appear seemingly random to the subject. For this test, the subjects received 10 s exposures to each level in the following order: Level 2 (25 mA, 50 Hz), Level 0 (0 mA, 0 Hz), Level 1 (12.5 mA, 25 Hz), Level 3 (37.5 mA, 75 Hz), Level 4 (50 mA, 100 Hz). Again, the countdown clock was played before each round of exposure, followed by a buzzer signifying the start of exposure at which point the research staff clicked play on the TENS device. Following the second buzzer after 10 s of exposure, the research staff turned the TENS off and subjects were again instructed via the screen to remain still for an additional 10 s of ramp-down data collection. This process was completed for each of the three levels, with a 30 s recovery period between each round.

### 2.5. Data Processing

Self-reported anxiety VAS scores were not always in line with observed subject reactions, potentially due to the military environment in which the data was collected as military personnel may be desensitized to pain anticipation anxiety or otherwise may not wish to express heightened anxiety levels [44]. Subjects who responded four or below for all anxiety VAS responses (corresponding to low or to no anxiety) were removed from the dataset as non-responders to reduce the impact of outliers.

EDA and PPG signals were sampled at 4 Hz and 50 Hz, respectively. Traditional EDA components of skin conductance level (SCL) and skin conductance response (SCR) were extracted from each entire continuous recording using the cvxEDA and SparsEDA methods [45,46]. Time–frequency measurements of TVSymp and MTVSymp were also extracted. PPG pulse locations were found using the Waveform Envelope Peak Detection (WEPD) method and manually corrected to remove false peaks [47]. These were then transformed into a continuous heart rate signal.

These continuous signals were partitioned to the duration of each anticipation and each stimulation segment. From each segment, extracted EDA features included mean and variance of SCL, the number of non-specific SCRs greater than a threshold 0.05 μS (NSSCR) as shown in Equation (1), linear SCL slope as defined by the Ordinary Least Squares slope of the SCL segment, Hjorth Activity, Mobility, and Complexity as shown in Equations (2)–(4) where x(t) is the filtered EDA signal, total EDA spectral power computed with Welch’s estimate of power spectral density between 0 and 2 Hz, EDASymp [27], mean and maximum TVSymp and MTVSymp [25,29], and slope to the maximum TVSymp and MTVSymp values computed from the peak of maximum peak to the preceding trough or elbow point. Time–frequency features were also normalized by total EDA spectral power to reduce between-subject variance. Extracted PPG features included mean heart rate (HR), HR range as shown in Equation (5), Peak-to-Peak Interval (PPI) standard deviation, and the PPI root mean squared of successive deviations as shown in Equation (6). Pain/stimulation segment features were calculated during the 10 s of stimulation, and anxiety segment features were calculated for the 10 s before stimulation.

(1) $$NSSCR=Count(SCR\left(t\right)\geq 0.05\;\&\;SCR\left(t-1\right)<0.05)$$

(2) Activity=var(x(t))

(3) $$Mobility=\sqrt{\frac{Activity(\frac{dx(t)}{dt})}{Activity(x(t))}},$$

(4) $$Complexity=\frac{Mobility(\frac{dx(t)}{dt})}{Mobility(x(t))},$$

(5) $$RangeHR=\max\left(HR\right)-min(HR)$$

(6) $$RMSSD=\sqrt{\frac{1}{N-1}\sum_{i=1}^{N-1}{\left(PPI(i+1)-PPI(i)\right)}^{2}}$$

All computed features are shown in Table 1. All features had a raw and a baseline-normalized version. A baseline value of each feature was obtained from a 10 s segment during the rest prior to the start of testing. All features had a raw value and a baseline-normalized value obtained by subtracting the subject baseline value from the raw value.

### 2.6. Machine Learning

Once all features were extracted, they were normalized using subject-wise standard scaling, i.e., subtracting the mean and dividing the result by the standard deviation to bring the majority of features within a 0 ± 2σ range personalized to each subject. Data processing and model training were conducted as follows: First, raw EDA and PPG signals were filtered, then subsignals were extracted (SCL, SCR, TVSymp, MTVSymp, PPG Peaks/PPI, HR). HR was calculated from PPI and interpolated to 4 Hz using PCHIP. These subsignals were then segmented to the duration of each trial recording (the anxiety segment before and the pain segment during the stimulation) and features extracted for each segment to obtain our feature set. A classification pipeline was constructed with a feature normalization block, followed by a feature selection step using recursive feature elimination, which iteratively removes the least informative features from model decisions if the resulting smaller model performs better, followed by a hyperparameter search step using a randomized grid search with successive halving, and finally the classification model. The pipeline was trained using leave-one-subject-out cross-validation (LOSO-CV). For each fold of the model cross-validation model, data from all but one subject were used to train the pipeline and then the resulting selected features and model were validated on the one left-out subject to evaluate model performance in a simulated subject-unseen real-world scenario. Due to the imbalance between target class sizes, we used balanced class accuracy (BAC, Equation (7)) as our optimization metric during the feature and hyperparameter searches and evaluated the model performance using BAC and area under the receiver operator characteristic curve (AUC).

(7) $$BAC=\frac{Sensitivity+Specificity}{2}$$

After model performance metrics were determined using the pipeline, feature selection and hyperparameter searching were each performed again in sequence using LOSO-CV to determine average optimal features and hyperparameters, then these were used to train a single final model. This final model was analyzed using SHapley Additive exPlanations (SHAP) to determine how each feature on average contributed to model classification [48]. Models investigated were: Xtreme Gradient Boosting (XGB), Support Vector Machine (SVM), Random Forest Classifier (RFC), Logistic Regression (LR), and K-Nearest Neighbors (KNN). This process was repeated for target scores of pain VAS and stimulation level for pain, previous pain, and previous stimulation level for anxiety.

## 3. Results

### 3.1. Statistics

Of the 82 subjects included in this study, 54 were male and 28 were female. Subject ages ranged from 18 to 85, with a median of 36 and an interquartile range of 17.5. The median and interquartile ranges of VAS scores are shown in Table 2.

VAS anxiety scores given by some subjects were often inconsistent with their observed actions. This visible inconsistency inspired doubt of the truth of the anxiety VAS scores. Instead, the pain score reported during the previous test was used as an analog ground truth for anxiety level, as the most recent painful stimulus was believed to impact the anticipation of the severity of the next painful stimulus. The resulting previous pain score measurement showed a high correlation with anxiety VAS scores (Pearson’s Correlation r = 0.65) while also having a larger spread of scores and more consistent agreement with observed anxiety responses.

After exclusions, 20 subjects were included in the final model dataset. Of this, 29% of all responses had medium or higher pain VAS scores (TGI: 8%, TENS: 45%).

### 3.2. Pain Modeling

Model performance metrics are shown below in Table 3. Logistic regression performed best on all pain VAS models across all metrics for all segment types, except for the linear SVM which returned higher balanced accuracy for TENS segments. Linear kernel SVMs performed best for all-segment stimulation scores and gave the highest balanced accuracy and second highest AUC on TENS segments behind the logistic regression model. Logistic regression performed the best on TGI segments. Models trained on all segment types returned higher pain VAS performance compared to either individual segment type; however, this trend does not hold for stimulation level where TENS segments achieved near-perfect classification. Stimulation level performance was generally higher than pain VAS performance, although top models for each segment type returned balanced accuracies above 80%.

**Figure 3:**
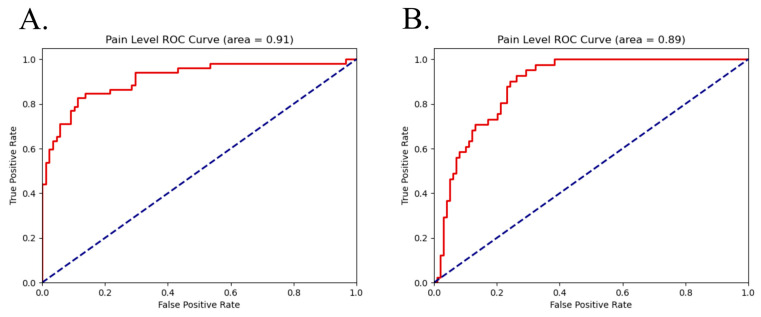
ROC curves (in red) for all segment classification for (**A**) pain using a logistic regression model and (**B**) stimulation using a support vector machine. Random classification ROC curve is shown in blue for comparison.

ROC curves for the top performing all-segment models are shown below in Figure 3.

**Figure 4:**
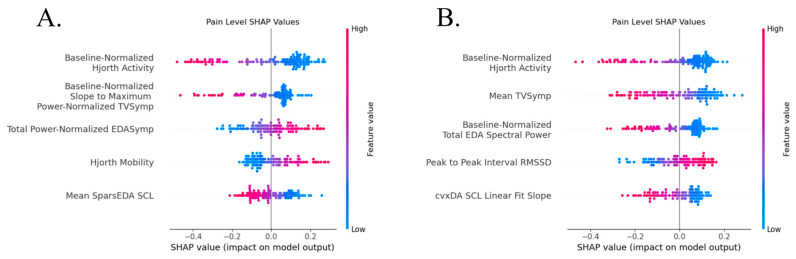
Most impactful features by SHAP values for all segment classification for (**A**) pain using a logistic regression model and (**B**) stimulation using a support vector machine.

The top five most impactful features on each model by Shapley values are shown below in Figure 4. The horizontal axis shows the impact on model prediction, with positive and negative values indicating a shift towards either class. Color shows the distribution of feature values between the two classifications. Pain classification for both target types was largely driven by Hjorth Activity, EDA spectral power (both EDASymp and EDA Total Spectral Power), and TVSymp-based metrics. Higher Hjorth Activity, mean TVSymp, and a steeper slope to TVSymp maximum all lead to the detection of pain. PRV-derived features contributed minorly to classification when stimulation level was the target. All top features show a monotonic relation between feature value and model classification, with high or low feature values uniformly pushing decisions in one direction or the other, and had a large range of feature values.

### 3.3. Anxiety Modeling

Model performance metrics are shown below in Table 4. Highest performance on each segment type for each balanced accuracy and AUC are bolded.

The linear kernel SVM performed best when trained on all segment types, though all models except the KNN returned balanced accuracies above 75%. The linear kernel SVM and the logistic regression models had values of 0.7773 and 0.7647, and 0.7994 and 0.8169, for balanced accuracy and AUC performance respectively, on TENS segments, which was lower than all-segment model performance. Best TGI balanced accuracy performance was achieved by the random forest classifier, though it also yielded a low AUC. The logistic regression model had the highest AUC and the second highest balanced accuracy. Previous pain VAS performance on TGI segments was above all-segment performance. Xtreme gradient boosting had the highest performance on both metrics for all segment types except for TGI segments, where logistic regression yielded a higher AUC. As with the stimulation models above, models trained on TENS segments alone produced higher performance than TGI segments with all-segment performance in between. All-segment previous pain performance was above all-segment previous stimulation performance.

**Figure 5:**
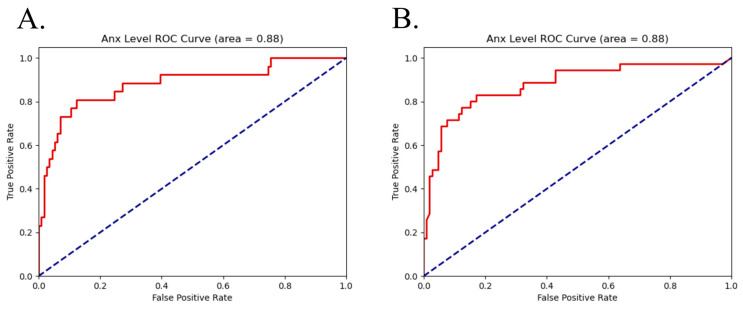
ROC curves (in red) for all-segment classification for previous pain (**A**) using a support vector machine and previous stimulation (**B**) using an Xtreme gradient boosting model. Random classification ROC curve is shown in blue for comparison.

ROC curves for the top performing all-segment models are shown below in Figure 5.

**Figure 6:**
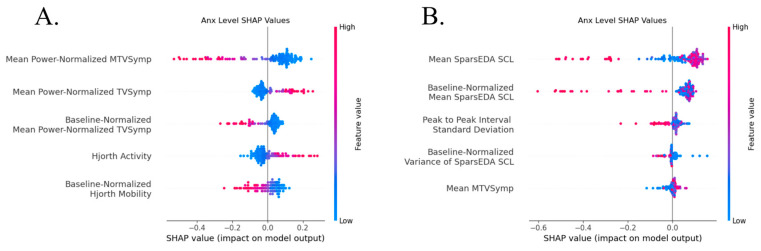
Most impactful features by SHAP values for all segment classification for (**A**) previous pain using a support vector machine and (**B**) previous stimulation using an Xtreme gradient boosting model.

The top five most impactful features on each model by Shapley values are shown below in Figure 6. Top anxiety classification features differed based on anxiety target type. Previous pain level-derived anxiety classification was driven by MTVSymp, TVSymp, and Hjorth Activity, with a higher mean power-normalized MTVSymp and mean power-normalized and baseline-normalized TVSymp indicating an anxious state. These features show a monotonic spread between feature value and SHAP impact, but with large clusters of low values indicating a higher impact from larger deviations. SCL dynamics and PRV features drove decisions in previous stimulation-level-derived anxiety classification, with higher SCL, baseline-normalized SCL, and PPI standard deviation indicating an anxious state. These features are not as monotonic, with low and medium feature values having similar classification impacts while high feature values drove most of the classification.

## 4. Discussion

VAS scoring is inherently subjective and is often shaped by individual experience, perception, and potentially by social expectations surrounding how one should respond to a given stimulus. The meaning of terms like “anxiety” may depend on situational context and sociopolitical background, meaning that VAS instruments must be carefully tailored to the target population and clinical context or risk producing biased results. By building a model based on the objective physiologically of how a body reacts to different situations, we can potentially increase the reliability and diagnostic usefulness of these measurements while still providing the same ease of use of VAS scoring methods.

Our dataset encompassed a wide range of adult ages, focused primarily on the working age group of 18 to 54, though only 34% of subjects were female. Physiological differences between sexes and known sociological differences in pain and anxiety response mean that this imbalance may limit the generalizability of the resulting models. This population was primarily drawn from both white-collar and military populations. We did not analyze the impact of race in this dataset.

Prior literature has well established the use of EDA and HRV for pain level classification, which these results fall in line with [49,50]. Both models trained on subject-supplied pain VAS scores and objective levels of stimulation yielded high degrees of classification accuracy. Stimulation-level performance on all segments was higher than pain VAS level, which suggests these measurements may be able to not only reflect perceived pain but may better represent the objective level of the stimulus that caused the pain.

For both VAS and stimulation intensity scoring methods, single stimulation type model classification performance was similar to performance of the model trained on both stimulation methods, implying a high degree of generalization for these measurements. Pain classification was driven largely by rapid increase in EDA signal in previously identified stress-targeted ranges. These changes, indicating a rapid increase in sympathetic response, showed a repeatable and monotonic relation between the size of the EDA change and the likelihood that it corresponded to a painful stimulus, suggesting that EDA is a useful method of detecting not just pain but also potentially the level of pain. PRV features were less informative, likely due to the slower rate of change in heart rhythms in response to stress and the short duration of the sensing window. These features were able to inform classification using linear models with minimal feature transformation. With the increasing calls for explainable machine learning in healthcare, this gives confidence in the reliability of the signals for interpretable and biologically informed modeling.

Anxiety modeling was hampered by the low prevalence of elevated anxiety VAS scores in the dataset, which reduced effective sample size. While in real world clinical settings most patients do not present with the highest level of pain or anxiety, the reduced range of anxiety presentation reduced modeling effectiveness and required imbalance-tolerant metrics such as balanced accuracy in our model training and evaluation to help accurately assess performance. Observed behavioral reactions frequently contradicted self-reported scores, including one case in which a participant withdrew from the study without ever recording an anxiety VAS above 1. Because direct anxiety self-report proved unreliable, we explored surrogate measures and found that previous pain VAS scores and previous stimulation levels both correlated strongly with anxiety VAS while also better reflecting observed anxiety behavior and providing a wider range for class stratification. Previous stimulation has served as a substitute for stress and anxiety scoring in previous studies [51]. Given that anxiety is known to heighten pain perception, pain VAS scores carry information about the subject’s anxiety state, even when that influence operates outside conscious awareness, making prior pain an informative and stable proxy. Models trained on these surrogates achieved high accuracy. Anxiety is a slower, more internally mediated process than pain and is therefore less purely stimulus-driven than pain, and so these proxies are not exact. As a potential result, each feature in the previous pain VAS score model had an overall lower SHAP impact. Anxiety classifications were largely driven by both EDA dynamics in previously identified stress-targeted ranges and slower-moving SCL dynamics. Since pain anticipation anxiety is a slower and more prolonged feeling than pain, SNS activity is expected to be higher than baseline and more stable than the rapid changes seen in response to pain. Due to the prolonged feeling of anxiety prior to pain, heart rhythms would have time to adapt to the SNS activity and PRV metrics could potentially be informative, though this was not observed in this study. Future research with expanded anxiety questionnaires and scoring methods are needed to truly evaluate how these features can translate to objective detection.

Motion artifacts represent a fundamental challenge in ambulatory EDA and PPG monitoring. EDA signals in particular are highly corruptible by motion due to variations in skin–electrode contact distorting the conduction pathway [52]. Depending on the type of motion, these artifacts can mimic non-artifact EDA responses and create false detections. In clinical settings, patient motion is often kept to a minimum; however, this cannot always guarantee a clean signal. Prior studies have been able to detect these motion artifacts automatically with a high degree of accuracy from ultra-short duration segments, but they still represent lost information in pain or anxiety detection. More work is needed to help improve detection of pain and anxiety in a potentially noisy clinical setting. Longer signal windows have also shown better results in classification tasks relying on biosignals, in one study improving cognitive load classification from 60% to 68% when the window length was increased from 5 to 25 s [53]. The natural latency in EDA response is up to 5 seconds post stimuli, and an SCR duration is 1–3 seconds on average, both of which combine to create a physiological limit how much information the body generates and can be collected from sensors within a small sampling window post-stimulus [54,55]. However, increasing the detection window duration delays clinician response to any pain or anxiety experienced by the patient, potentially leading to unnecessary suffering that could have otherwise been caught. Additional signal modalities such as electromyography (EMG) or skin sympathetic nerve activity (SKNA) may help address this trade-off.

The reduced dataset size impacted the ability to perform multiclass modeling and to use more advanced deep learning techniques, which often require more samples to generalize. Available public datasets differ in the reporting and reliability of their pain scoring and anxiety scoring and are widely varying in what type of anxiety they attempted to detect. Due to the differences in physiological presentation between different types of anxiety, public datasets collected to investigate social or general anxiety were not able to be used to amplify our training dataset. Additionally, context has been shown to be important for proper anxiety detection, further complicating the use of public datasets. Future studies with better control of anxiety reporting and larger sample sizes may open the door to these techniques and further pave the way towards a more accurate and more generalized clinic-ready model.

Anxiety comes in many forms, including generalized anxiety, social anxiety, specific phobias, or any of a multitude of other anxiety forms [56]. In this study, we only evaluated pain anticipation anxiety as that is the primary anxiety form indicated in surgical environments. Prior research has found that anxiety detection performance improves if context is provided to the model (i.e., the circumstances in which biosignals are collected that may indicate what arousal is being driven by), and thus building this context-dependent model helps to target this specific use case [39]. We will need further research into EDA in other anxiety-inducing situations to increase the usability of these models in additional contexts.

## 5. Conclusions

Using no more than 10 s of wearable sensor measurement, we were able to accurately classify both pain and anticipation anxiety with a high degree of accuracy. We were able to verify these models on two different forms of stimulation to increase real-world generalizability using both subjective and objective measures. Features selected by these models show smooth and continuous distribution on prediction impact, providing both insight into the physical bodily changes occurring and explainability to care providers. However, our dataset was limited by a skew towards low pain and low anxiety scores. Additional stimulation methods, a wider population sample, and more segments will help to build a truly generalized model.

## Figures and Tables

**Table 1:** List of extracted features for machine learning.

Signal	Type	Features
EDA	Time-domain	NSSCR (EDA), mean, maximum (SCL, TVSymp, MTVSymp, TVSymp_n_ MTVSymp_n_)
	Trend-based	Linear Slope (SCL), Slope to Peak Amplitude (SCL, TVSymp, MTVSymp)
	Frequency-domain	Total EDA Power, EDASymp, EDASymp_n_
	Hjorth parameters	Activity, Mobility, Complexity
PRV		PPI s.d., PPI RMSSD, mean HR, HR Range

**Table 2:** Median and interquartile range of reported pain and anxiety VAS scores for TGI and TENS.

		TGI 0	TGI 1	TGI 2	TENS 0	TENS 1	TENS 2	TENS 3	TENS 4
Pain VAS Scores	Median	0	1	2	0	2	3	5	6
	Interquartile Range	0	2	3	0	3	3	4	4
Anxiety VAS Scores	Median	1	0	0	1	1	1	0	1
	Interquartile Range	2	1	1	2	3	2	2	3

**Table 3:** Balanced accuracy and area under the ROC curve for all pain and stimulation models and segment types.

		Pain VAS		Stimulation Level	
Segment Type	Model	Balanced Accuracy	AUC	Balanced Accuracy	AUC
All	XGB	0.815	0.879	0.839	0.878
	SVM	0.775	0.809	**0.857**	**0.914**
	RFC	0.813	0.858	0.791	0.838
	LR	**0.832**	**0.889**	0.833	0.875
	KNN	0.770	0.776	0.765	0.809
TENS	XGB	0.780	0.756	0.984	0.986
	SVM	**0.809**	0.815	**0.990**	0.995
	RFC	0.698	0.722	0.974	0.986
	LR	0.807	**0.847**	0.984	**0.999**
	KNN	0.679	0.697	0.932	0.981
TGI	XGB	0.691	0.629	0.725	0.694
	SVM	0.500	0.891	0.688	0.649
	RFC	0.664	0.629	0.663	0.688
	LR	**0.746**	**0.756**	**0.738**	**0.781**
	KNN	0.500	0.500	0.700	0.674

The best performing model by each metric is bolded.

**Table 4:** Balanced accuracy and area under the ROC curve for all previous pain and previous stimulation models and segment types.

		Prev. Pain VAS		Prev. Stimulation Level	
Segment Type	Model	Balanced Accuracy	AUC	Balanced Accuracy	AUC
All	XGB	0.8202	0.8431	**0.8286**	**0.8767**
	SVM	**0.8424**	**0.8809**	0.7000	0.7252
	RFC	0.7848	0.7932	0.7952	0.8014
	LR	0.8249	0.8428	0.7714	0.7671
	KNN	0.6613	0.6501	0.6524	0.6493
TENS	XGB	0.6106	0.6064	**0.8777**	**0.9419**
	SVM	**0.7773**	0.7994	0.7607	0.7643
	RFC	0.6205	0.5934	0.8624	0.8947
	LR	0.7647	**0.8169**	0.8200	0.8257
	KNN	0.7296	0.6137	0.6622	0.7090
TGI	XGB	0.6667	0.6111	**0.8137**	0.7772
	SVM	0.8158	0.8304	0.7733	0.7873
	RFC	**0.8715**	0.6199	0.7919	0.7919
	LR	0.8684	**0.8830**	0.7624	**0.8059**
	KNN	0.5000	0.5000	0.6040	0.6250

The best performing model by each metric is bolded.

## Data Availability

The data presented in this study are available on request from the corresponding author.

## Disclaimer

The views expressed in this article reflect the results of research conducted by the authors and do not necessarily reflect the official policy of the Department of the Navy, Department of Defense, nor the United States Government. Some authors are military service members and employees of the United States Government. This work was prepared as part of their official duties. Title 17 U.S.C. §105 provides that “Copyright protection under this title is not available for any work of the United States Government.” Title 17 U.S.C. §101 defines a U.S. Government work as a work prepared by a military service member or employee of the U.S. Government as part of that person’s official duties.
